# In-Gel Determination of L-Amino Acid Oxidase Activity Based on the Visualization of Prussian Blue-Forming Reaction

**DOI:** 10.1371/journal.pone.0055548

**Published:** 2013-02-01

**Authors:** Zhiliang Yu, Ning Zhou, Chuntian Zhao, Juanping Qiu

**Affiliations:** College of Biological and Environmental Engineering, Zhejiang University of Technology, Hangzhou, China; Instituto Butantan, Brazil

## Abstract

L-amino acid oxidase (LAAO) is attracting increasing attention due to its important functions. Diverse detection methods with their own properties have been developed for characterization of LAAO. In the present study, a simple, rapid, sensitive, cost-effective and reproducible method for quantitative in-gel determination of LAAO activity based on the visualization of Prussian blue-forming reaction is described. Coupled with SDS-PAGE, this Prussian blue agar assay can be directly used to determine the numbers and approximate molecular weights of LAAO in one step, allowing straightforward application for purification and sequence identification of LAAO from diverse samples.

## Introduction

All the described L-amino acid oxidases (LAAOs; EC 1.4.3.2) are flavoenzymes, except the lysine oxidase of *Marinomonas mediterranea*, catalysing the stereospecific oxidative deamination of L-amino acids to the corresponding α-keto acids with the release of ammonium and H_2_O_2_ [1], [2]. This enzyme is widely distributed not only in snake and insect venoms [3], but in sea hare [4], fungi [5], bacteria [6], [7], and algae [8]. So far snake venom LAAO is the best investigated member of this enzyme family with respect to not only toxicology but also biochemistry, physiology and medicine. LAAO is attracting more attentions due to its important biological roles, such as anti-microbial, anti-insect, anti-tumor cell, consumption of amino acids, etc. It is believed that its biological roles are probably associated with the produced H_2_O_2_ [6], [9].

While a range of methods are applicable to the detection of LAAO activity, including measurement of production, such as ammonia [10], α-keto acid [11] and H_2_O_2_ [12], measurement of oxygen consumption using a classic Warburg manometer or an oxygen-sensitive electrode [13] and measurement of amino acid substrate change [14], each has its own advantages and disadvantages. Among these methods, the detection methods for measurement of H_2_O_2_ production constitute attractive assays. In particular, the horseradish peroxidase (HRP) which acts as H_2_O_2_ sensitive probes is the most widely used for the detection of H_2_O_2_ due to its reasonable simpleness, sensitivity and reproducibility [15]. However, most of the HRP substrates, including 2,2′-azino-bis(3-ethylbenzthiazoline-6) sulphonic acid (ABTS), o-phenylendiamine and o-dianisidine are mutagenic, carcinogenic or extremely toxic compounds, and HRP itself is easily inactivated and expensive. These defects may limit its further applications.

Prussian blue has been known since 1704, and has been definitely one of the most ancient coordination materials and an important pigment for color uses [16]. As an advanced transducer, it has been adapted to sensors for detection of H_2_O_2_ and other easily oxidizable compounds. It was also applied to biosensors for transition of metal hexacyanoferrate [16]. Scientists have denoted it as an “artificial peroxidase” on account of its high activity and selectivity, which are commonly the properties of biocatalysis. These properties of Prussian blue as artificial peroxidase provide the possibility for determination of LAAO activity by detecting the produced H_2_O_2_ based on the visualization of Prussian blue-forming reaction. Recently, Prussian blue agar medium assay for the detection of H_2_O_2_ was preliminarily reported to screen the LAAO activity [17], [18]. The purpose of the present study is to describe a new application of Prussian blue for quantitative in-gel determination of the presence of LAAO activity by detecting the produced H_2_O_2_ concentration. Compared with HRP assay, Prussian blue agar assay shows the benefits, including more simpleness, cost-effectiveness and convenience. Another key advantage of Prussian blue assay over HRP assay is that Prussian blue assay can be directly used for in-gel determination of the numbers and molecular weights of LAAO on the coupled SDS-PAGE after visualization of Prussian blue-forming reaction.

## Results

Prussian blue assay has been widely used in sensors to detect the presence of H_2_O_2_ in electrochemical reactions because iron (III) and potassium hexacyanoferrate (III) can be oxidized to yield the blue precipitate of Prussian blue by the sequential reactions as shown below. It should be noted that in this assay H_2_O_2_ acts as electron donor.
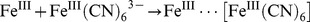


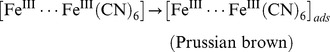


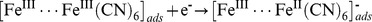


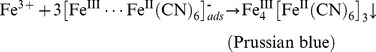
This color change is fast, sensitive and reproducible. In the present study, we used normal marine medium (MM) to prepare the plates for Prussian blue assay and adjusted the pH to around 7.5 before autoclave. Usually, after pouring, the agar plates gave Berlin green, which was caused by the following reactions:
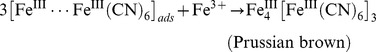


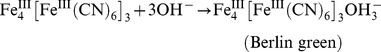
We found that the pH adjustment step or addition of NaOH during assay medium fabrication is critical to the resultant color of agar plate. Slight pH change of Prussian blue agar medium after adjustment before autoclave could cause slight color difference of resultant agar plate after pouring. Probably, Prussian blue is a complicate class of chemical compounds and it contains Prussian blue, Prussian brown, Prussian white and Berlin green. But it should be pointed out that in our Prussian blue assay, slight color difference of resultant agar plate before assay due to oscillation of adjusted pH around 7.5 did not affect the color mature of Prussian blue caused by H_2_O_2_. In addition, it was found that without peptone and yeast extract the resultant Prussian blue agar plate was opaque and greenish-white in color and had also no effect on color mature of Prussian blue.

**Figure 1:**
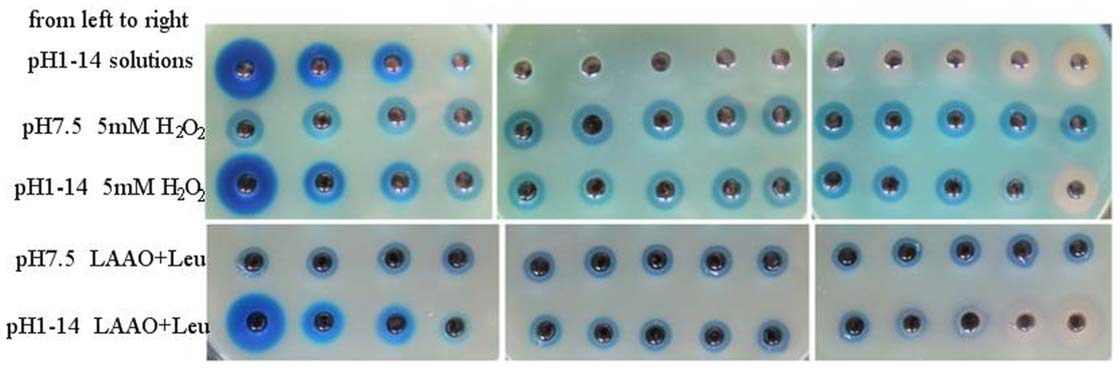
Background pH dependence of Prussian blue agar assay for detection of H_2_O_2_. Row 1 from left to right: pH 1 up to 14 of background solutions prepared by a mixture of 6 N HCl with 6 N NaOH; row 2: individual standard 5 mM H_2_O_2_ solutions with uniform pH of 7.5; row 3 from left to right: individual standard 5 mM H_2_O_2_ solutions with different pH values ranging from 1 to 14 adjusted by either HCl or NaOH; row 4: the detection of oxidization reactions of L-Leu by LAAO harvested from strain R3 with uniform pH of 7.5; row 5 from left to right: same as row 4, except that the pH values of reaction solutions were adjusted to 1 to 14 after oxidation. Prussian blue agar medium contained peptone and yeast extract in the upper three rows, but no peptone and yeast extract in the lower two rows.

Considering the fact that the color of resultant Prussian blue agar medium is sensitive to the medium pH, we investigated the effect of detection solution pH on the color development of Prussian blue agar. First, we mixed 6 N HCl with 6 N NaOH to prepare background solutions with different pH values ranging from 1 to 14 for Prussian blue agar test. As shown in row 1 of Figure 1, background solutions with pH from 5 to 9 almost did not cause any color change of Prussian blue agar which remained original Berlin green. However, either lower pH or higher pH did make the color change of plate. The background solutions with pH from 1 to 4 all gave brilliant Prussian blue holes, probably due to the degradation of partial hexacyanoferrate under peracid condition to release CN^−^, followed by the reduction of Fe^3+^ to Fe^2+^ by CN^−^ and subsequent formation of Prussian blue (either “water-insoluble Prussian blue” Fe^III^_4_[Fe^II^(CN)_6_]_3_ or “water-soluble Prussian blue” KFe^III^Fe^II^(CN)_6_). In addition, the bigger diameters of Prussian blue holes were generated by the lower pH of background solution, probably due to the stronger degradation of hexacyanoferrate. On the other hand, the background solutions with pH values from 10 to 14 all generated fade-white holes, probably due to the white precipitation of Fe(OH)_3_. Next, the standard 5 mM H_2_O_2_ solutions with the same pH of 7.5 were prepared for Prussian blue agar test. As indicated in row 2, all standard H_2_O_2_ solutions presented uniform and reproducible blue holes with almost the same size. In contrast, the 5 mM H_2_O_2_ solutions with different pH values ranging from 1 to 14 yielded different resultant blue holes (row 3 in Figure 1). Both peracid (pH 1∼3) and peralkaline (pH 13∼14) had strong influence on the color development of Prussian blue agar under H_2_O_2_ pressure, while the conditions with pH values from 4 to 12 had no significant influence on the formation of Prussian blue holes by H_2_O_2_. Similarly, as expected, the oxidization reactions of L-Leu by LAAO from the strain R3, an LAAO-producer, with the same pH of 7.5 all gave uniform and reproducible blue holes caused by the released H_2_O_2_ (row 4 in Figure 1). However, after oxidation reaction of L-Leu by LAAO, the adjustment of pH to 1∼14 with either HCl or NaOH yielded different blue holes. The pH of oxidization solution adjusted to either 13 or 14 completely inhibited the formation of Prussian blue under the released H_2_O_2_ produced by LAAO activity. In contrast, the adjusted pH ranging from 5 to 10 had no obvious inhibition. Again, peracid detection condition with pH 1∼4 caused serious degradation of hexacyanoferrate and formed very big blue holes. Moreover, the assay pH of either 11 or 12 showed slight inhibition of the formation of Prussian blue. All these findings indicate that the pH value of detection solution suitable for Prussian blue agar assay is around 5 up to 12 and the best one is 7 up to 10. In the following study, we used around 7.5 as the detection solution pH.

**Figure 2:**
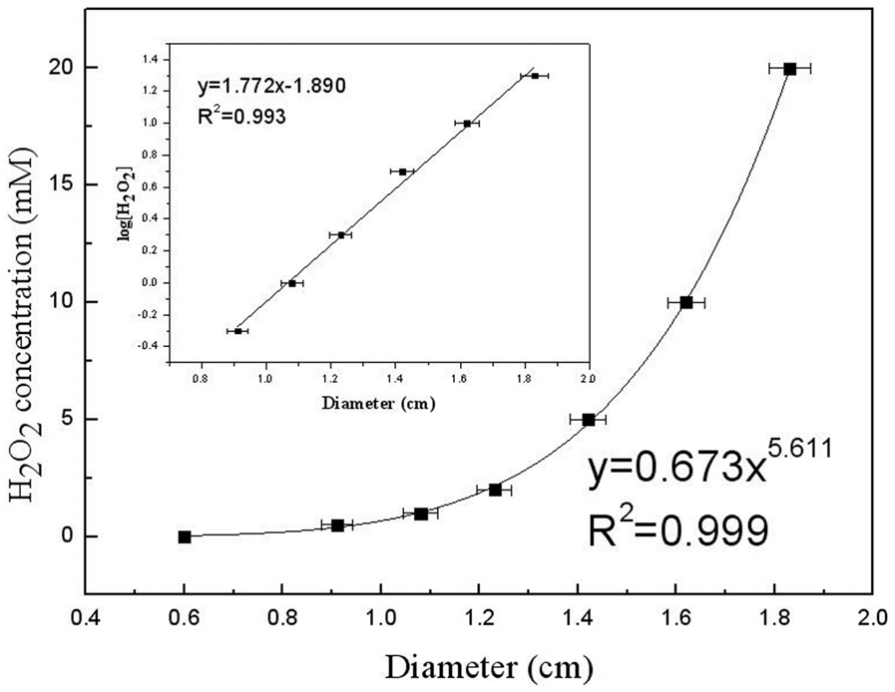
Correlation between the detected H_2_O_2_ concentration and the diameters of Prussian blue holes. The distribution can perfectly be fitted with the exponential equation y = 0.673×^5.611^, where x is the diameter of blue hole and y the H_2_O_2_ concentration. The inset showed that in the range of 0.5 mM≤H_2_O_2_≤20 mM, the change in diameter of blue hole was a function of logarithm of H_2_O_2_ concentration with linear fits under the equation y = 1.772x–1.890, where x is the diameter of blue hole and y the logarithm of H_2_O_2_ concentration.

**Figure 3:**
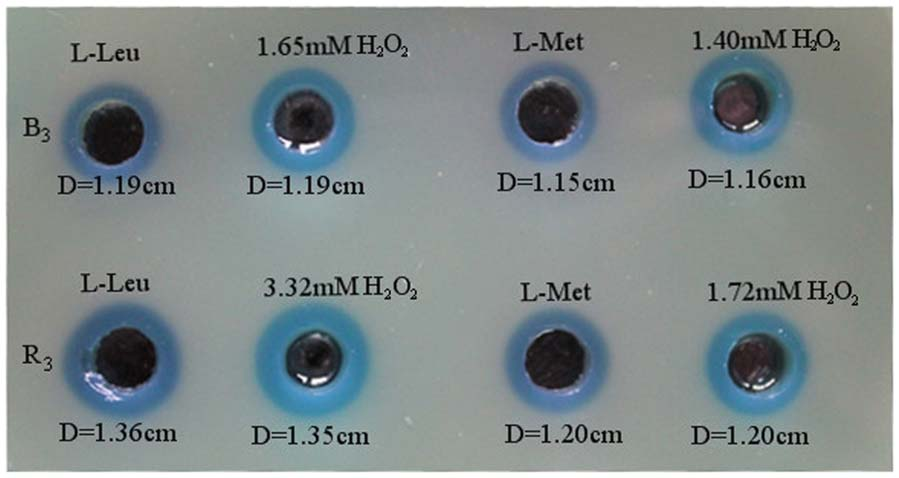
Measurement of LAAO activities produced by *Pseudoalteromonas* sp. B3 (upper) and *Pseudoalteromonas* sp. R3 (lower) treated with different amino acids, L-Leu and L-Met as indicated above the corresponding holes. On the basis of the diameters of the formed Prussian blue, the concentrations of H_2_O_2_ produced by LAAO activities were calculated by using the equations in Figure 2. The corresponding standard H_2_O_2_ solutions as indicated above the corresponding holes were used to confirm the accuracy of Prussian blue agar assay for detection of LAAO activity. The diameters of the blue holes were indicated under the holes.

To investigate the sensitivity of Prussian blue agar to H_2_O_2_, a series of pH 7.5 H_2_O_2_ solutions with different concentrations ranging from 0.5 mM to 30 mM were prepared for Prussian blue agar assay. As displayed in Table 1, all the tested H_2_O_2_ gave Prussian blue holes. The higher the H_2_O_2_ concentrations from 0.5 mM to 20 mM, the bigger the diameters of blue holes, indicating that Prussian blue agar assay is sensitive to 0.5 mM≤H_2_O_2_≤20 mM, while further increases of H_2_O_2_ concentration to 25 mM and even 30 mM did not obviously make the blue holes bigger, probably due to the saturation of Prussian blue agar to H_2_O_2_. The statistical analysis of mean difference of blue hole diameters from different H_2_O_2_ showed that the increase of hole diameters is extremely significant (P<0.001) with the increase of H_2_O_2_ from 0 to 20 mM, but not significant (P>0.05) with the increase of H_2_O_2_ from 20 mM to 30 mM (Table S1). To push the detection limit, the Prussian blue agar was used to detect the H_2_O_2_ with concentrations from 100 µM to 500 µM. As displayed in Figure S1, unlike the CK (background solution, without H_2_O_2_), all the H_2_O_2_ solutions with different concentrations gave blue holes. The diameters of blue holes expectedly increased with the increase of H_2_O_2_ concentration. However, it was difficult to very accurately measure the diameters of blue holes caused by H_2_O_2_≤400 µM. To observe the correlation between H_2_O_2_ concentration from 0 to 20 mM and Prussian blue diameter, the data in Table 1 were plotted as shown in Figure 2. The distribution can perfectly be fitted with the exponential equation y = 0.673×^5.611^, where x is the diameter of blue hole and y the H_2_O_2_ concentration. Further plotting in Figure 2 inset indicated that in the range of 0.5 mM≤H_2_O_2_≤20 mM the change in diameter of blue hole was a function of logarithm of H_2_O_2_ concentration with linear fits under the equation y = 1.772x–1.890, where x is the diameter of blue hole and y the logarithm of H_2_O_2_ concentration. To estimate the LAAO activity described as H_2_O_2_ concentration fashion using the above extracted equations, the culture supernatants with different LAAO activities from strain B3 and strain R3, two LAAO-producers, were used to oxidize the substrates L-Leu and L-Met, respectively, in separate reactions, followed by Prussian blue agar (without peptone and yeast extract) assay. The results in Figure 3 showed that B3-LAAO with L-Leu and L-Met as substrates can generate Prussian blue holes with diameters of 1.19 cm and 1.15 cm, respectively, which correspond to the H_2_O_2_ concentrations of 1.65 mM and 1.40 mM, respectively, based on the above extracted equations in Figure 2. Similarly, R3-LAAO with L-Leu and L-Met as substrates can generate Prussian blue holes with diameters of 1.36 cm and 1.20 cm, respectively, which correspond to the H_2_O_2_ concentrations of 3.32 mM and 1.72 mM, respectively. To confirm the reliability of fitted curves or equations, the standard H_2_O_2_ with concentrations of 1.65 mM, 1.40 mM, 3.32 mM and 1.72 mM were used for the assay and finally gave the blue holes with diameters of 1.19 cm, 1.16 cm, 1.35 cm and 1.20 cm, respectively, all perfectly agreeing with our calculated concentrations based on the fitted equations. Therefore, the LAAO activity of B3 supernatant with L-Leu and L-Met as substrates had 0.330 U/ml and 0.280 U/ml, respectively, whereas the LAAO activity of R3 supernatant with L-Leu and L-Met as substrates had 0.664 U/ml and 0.344 U/ml, respectively, indicating that R3 supernatant has higher LAAO activity than B3. All these findings indicate that Prussian blue agar assay is feasible to distinguish the LAAO activity. Moreover, the extracted equations in the present study are reliable to quantitatively determine the LAAO activity. To confirm the method with another enzyme source using a purified enzyme, the commercial *Crotalus adamanteus* venom LAAO (caLAAO, Worthington Biochemical Corporation, USA) was applied to Prussian blue assay with L-Leu as substrate. Figure S2 showed that caLAAO gave a blue hole with 1.51 cm diameter which corresponds to around 6.11 mM H_2_O_2_ based on the extracted equation. When 6.11 mM standard H_2_O_2_ was applied to Prussian blue assay, a blue hole with 1.50 cm diameter appeared, indicating great reliability of Prussian blue assay for determination of LAAO activity.

**Table 1:** Measurement of the diameters of the blue holes.

H_2_O_2_/mM	0	0.5	1	2	5	10	20	25	30
blue halos diameters/cm	0.6	0.91±0.03	1.08±0.03	1.23±0.04	1.42±0.04	1.62±0.04	1.83±0.04	1.83±0.04	1.84±0.04

**Figure 4:**
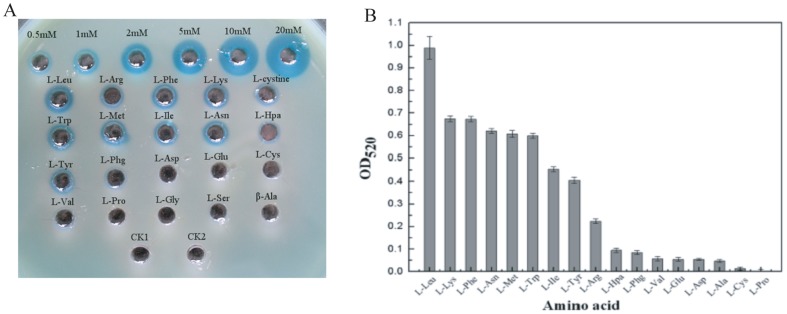
Characterization of substrate specificity of LAAO produced by *Pseudoalteromonas* sp. R3 based on Prussian blue agar assay (A) and 2, 4-dinitrophenylhydrazine (DNP) method (B). For Prussian blue agar assay, standard H_2_O_2_ solutions with different concentrations ranging from 0.5 mM to 20 mM were used as the positive controls; as the negative controls, CK1 and CK2 were the R3 culture supernatant without any substrate for oxidization. 2, 4-dinitrophenylhydrazine (DNP) method was applied to detecting α-keto acids derived from their corresponding L-amino acids by LAAO activity.

Each LAAO has its own substrate specificity. To investigate the substrate specificity of LAAO from R3, 20 kinds of common amino acids were selected as substrates for Prussian blue agar (without peptone and yeast extract) assay. It was found in Figure 4A that like positive controls of H_2_O_2_ with different concentrations from 0.5 mM to 20 mM, after oxidation by R3-LAAO, L-Leu, L-Lys, L-Phe, L-Asn, L-Trp, L-Met, L-Arg, L-Ile, L-Tyr, L-cystine, L-Hpa, L-Phg and L-Val yielded blue holes. The diameters of blue holes further showed that R3-LAAO had different activities to different substrates with an order of L-Leu>L-Lys≈L-Phe>L-Asn≈L-Met≈L-Trp>L-Ile≈L-Arg>L-Tyr≈L-cystine>L-Hpa≈L-Phg>L-Val, an overall agreement with the results obtained from another detection method using 2, 4-dinitrophenylhydrazine (DNP) which can react with carbonyl group of a-keto acids derived from corresponding amino acids to generate dinitro-phenylhydrazone with a brown-red color (Figure 4B). On the other hand, like negative controls of R3 supernatant (CK1 and CK2) without any substrate, other substrates mixed with R3 supernatant did not create clear blue holes, meaning no obvious oxidization activity. These findings indicate that Prussian blue agar assay can be used to determine the substrate specificity of LAAO.

A simple and sensitive method for in-gel determination of LAAO numbers and their approximate molecular weights is highly promising. To achieve this purpose, we tried to develop an SDS-PAGE coupled Prussian blue assay. First, the harvested supernatant from *Pseudoalteromonas* sp. R3 was precipitated by adding ammonium sulfate to 40% saturation. After centrifugation, the supernatant and precipitate, namely 40% supernatant and 40% precipitate, respectively, were collected for Prussian blue agar assay. As shown in Figure S3, both 40% supernatant and 40% precipitate gave blue holes, but 40% supernatant yielded more brilliant blue color. Therefore, the 40% supernatant was further precipitated by adding extra ammonium sulfate to 60% saturation. After another centrifugation, the supernatant and precipitate, namely 60% supernatant and 60% precipitate, respectively, were also collected for Prussian blue agar assay. It was found that 60% supernatant and 60% precipitate both yielded blue holes, but 60% precipitate presented more brilliant blue color. Therefore, Prussian blue agar assay can be used to track the LAAO activity during purification and separation. To determine the numbers and molecular weights of LAAO secreted by strain R3, the 60% precipitate without heating to maintain LAAO activity was electrophoresed on SDS-PAGE. After electrophoresis, the different lanes of SDS-PAGE were sliced out for different treatments and subsequently put together on Prussian blue agar. The results in Figure S4 showed that the duplicate sample lanes (lanes 2 and 3) without Coomassie brilliant blue (CBB) staining were directly put on Prussian blue agar and clearly appeared a blue band, indicating that strain R3 bears LAAO activity. Similarly, another sample lane (lane 4) with CBB staining also gave a clear Prussian blue band at the same position, which reveals that the LAAO used in this assay is resistant to SDS and ß-mercaptoethanol. To determine which protein band bears LAAO activity, all the CBB-stained protein bands near Prussian blue band were sliced out from a lane-4 replicate lane with CBB staining, and directly put on Prussian blue agar (lane 5). It was found that even after having been long time exposed to multiple steps treatment, the sliced target protein band with LAAO activity still clearly yielded a blue band, indicating that LAAO is very stable in this SDS-PAGE coupled Prussian blue agar assay. Given the molecular weights of CBB-stained standard protein marker (lane M) and control protein BSA (lane 1), the corresponding protein band with LAAO activity from strain R3 was estimated to be around 65 kDa in size on SDS-PAGE. To confirm this in-gel determination method for LAAO, caLAAO with different treatments was also subjected to SDS-PAGE coupled Prussian blue agar assay (Figure S5). Like R3-LAAO without CBB staining (lane 1), caLAAO without CBB staining (lane 2) also gave a clear Prussian blue band. Another caLAAO sample lane (lane 3) still remained the oxidization activity to form a Prussian blue band after CBB staining, indicating that caLAAO is also resistant to SDS and ß-mercaptoethanol in this assay. After release from a lane-3 replicate lane with CBB staining, the target protein band (lane 4) still generated a Prussian blue band. Results in Figure S5 showed that caLAAO had only one active protein band and its molecular weight was larger than one of R3-LAAO. All these findings indicated that SDS-PAGE coupled Prussian blue agar assay is friendly to determine the LAAO numbers and their approximate molecular weights.

## Discussion

In this communication, we demonstrated that Prussian blue agar assay can be used to detect the standard H_2_O_2_ solutions or H_2_O_2_ produced by LAAO activity. The diameters of the formed blue holes is perfectly a function of H_2_O_2_ concentration with exponential fits or logarithm of H_2_O_2_ concentration with linear fits varying from 0.5 mM to 20 mM, thus making Prussian blue agar assay quantitatively determine the LAAO activity as H_2_O_2_ concentration based fashion. Moreover, coupled with SDS-PAGE, Prussian blue agar assay can be directly used to not only sense the presence of LAAO activity, but also determine the numbers and approximately molecular weights of the LAAO protein in one assay, showing significant advantages in comparison to usual spectrophotometric and fluorometric detection methods for LAAOs.

The pH of detection solution is a critical factor to Prussian blue agar assay. There is no significant influence of detection solutions with pH 5 up to 12 on color development of Prussian blue agar. However, it was not recommended to use this assay under pH either below 4 or above 13 because both situations can shield the coloration of Prussian blue. Most probably, the peracid condition can cause the degradation of hexacyanoferrate, while peralkaline situation will make Fe^3+^ precipitation. Fortunately, common fermentation solutions produced by microorganisms or direct enzymatic reaction solutions have pH values in the range of 4∼12, thus giving broad applicability of this method.

When using Prussian blue agar assay, we found that prior to autoclave it was a key step to adjust the pH of Prussian blue agar solution to alkaline below 13 by adding NaOH. Otherwise, the Prussian blue agar plate will be very sensitive to the pH of detection solution. Most probably, NaOH can render Prussian blue agar buffering capacity. The key components of Prussian blue agar are iron (III) and hexacyanoferrate (III). Actually, these two chemicals are kind of flexible to mix with a variety of mediums, thus allowing wide applicability of this Prussian blue assay to screen LAAO-producing microorganisms. To detect the standard H_2_O_2_ solutions or H_2_O_2_ already produced by LAAO activity using Prussian blue agar assay, it is unnecessary to add peptone, yeast extract, etc. The simplest way to make Prussian blue agar plate is just to mix agar with iron (III) and hexacyanoferrate (III). However, to directly determine LAAO activity without oxidation reaction beforehand, it is necessary to supply substrates for LAAO, such as amino acid and peptone, into Prussian blue agar. Therefore, LAAO can oxidize its substrates present in Prussian blue agar to release H_2_O_2_ and trigger the coloration of Prussian blue agar. Similarly, to screen the LAAO-producing microorganisms, it is also necessary to add substrates of LAAO to Prussian blue agar plate.

Our results showed that the diameters of Prussian blue holes had clear correlation with detected H_2_O_2_ ranging from 0.5 mM to 20 mM in concentration. In fact, H_2_O_2_ with concentration of around 100 µM still can cause the formation of Prussian blue holes (Figure S1). But it is difficult to very finely measure the diameters of blue holes produced by 100 µM H_2_O_2_ because they were quite close to 6 mm, the diameter of fabricated circular well. Undoubtedly, this Prussian blue agar assay can detect the presence of standard H_2_O_2_ solutions or H_2_O_2_ produced by LAAO activity with concentration≥100 µM. In general, the LAAOs from different organisms have oxidation activities to release H_2_O_2_ with concentrations present in our detection level [19], [20], making our Prussian blue agar method direct application to sense the LAAOs activities. To quantitatively determine H_2_O_2_ with concentration>20 mM in this assay, the detection solution needs to be properly diluted before assay. This Prussian blue agar plate can be used to save a lot of workload for screening the LAAO-producing microorganisms from environmental samples. More importantly, it can be adapted to screening the mutants with different LAAO activities from the mutant library with altered expression of LAAO constructed by mutagenesis. Therefore, it can be applied to investigate the involved regulation mechanisms underlying the LAAO production, which is on the process in our lab.

The LAAO used in this study was resistant to SDS and ß-mercaptoethanol, and still kept its activity even after having been exposed to the staining procedure with CBB and destaining solution with glacial acetic acid. Therefore, the SDS-PAGE could be coupled with Prussian blue agar assay under non-denaturing conditions (without heating) to determine the numbers of LAAO and estimate its approximate molecular weights, showing clear advantages in comparison to commonly used spectrophotometric and fluorometric LAAO detection methods. Knowing directly the numbers and molecular weights of LAAO can be very helpful for further purification and characterization of this enzyme. In particular, the sliced target bands with LAAO activity could be directly analyzed with different techniques, such as protein sequencing and LC-MS/MS analysis.

A variety of methods have been developed to detect LAAO activity. Among them, the spectrophotometic and fluorometric methods are the most widely applied to detect LAAO activity through HRP involved reactions. A spectrophotometric 96-well microtiter plate is suitable for processing of large numbers of samples, although fluorometric measurement is more sensitive than spectrophotometric measurement. In many laboratories absorption-detecting micro-plate readers are more available than fluorescence-detecting ones. In these spectrophotometric assays, although HRP is hydrogen peroxide sensitive probes, it might be easily inactivated, unstable and expensive. Moreover, most of the HRP substrates are mutagenic, carcinogenic or extremely toxic compounds. In contrast, our Prussian blue agar assay doesn't need to rely on any detection instrument and can detect a large number of samples in the same reaction condition. Other advantages of our assay over spectrophotometic and fluorometric methods include convenient preparation, long-term storage at room temperature, simple operation and feasibility for direct determination of LAAO numbers and molecular weights. Rau et al. [15] also reported a simple, sensitive, rapid and reproducible in-gel detection method for LAAO, but their polyacrylamide gel containing LAAO needed to be soaked into the detection assay mixture containing HRP for coloration. Therefore, their method was relatively complex and expensive, and needed to be used right after it was ready. Besides, their polyacrylamide gel will appear visible smear, if the highly concentrated samples are used. To get sharply bounded bands, it seems to be necessary to carefully lower the LAAO concentration for their method. Although it is promising to further lower the detection limit, our in-gel detection method is more simple, cost-effective, stable, friendly and reproducible. It can be used for quantitative in-gel determination of the presence of LAAOs from the diverse samples, allowing broad and straightforward applications for screening the microorganisms after recombination or mutagenesis treatment.

## Materials and Methods

### Bacterial strain and culture condition

A yellow-pigmented LAAO-producing bacterial strain, designated as *Pseudoalteromonas* sp. B3, was isolated from the intertidal zone sludge sample (30.03°N, 122.11°E) located at Dinghai, Zhoushan, China. Phylogenetic analysis of 16S rDNA indicated that it belongs to the genus *Pseudoalteromanas* and its closest neighbor is the strain of *Pseudoalteromonas viridi* (98.5% identity). Previous detections of the produced α-keto acids [11], NH_4_^+^ (pH paper method) and H_2_O_2_ (Amplex Red Hydrogen Peroxide/Peroxidase Assay kit, Invitrogen, USA) all revealed that marine bacterial B3 can produce LAAO (data not shown). The pH paper method for detection of NH_4_^+^ was performed as follows: 3 ml of 1 M NaOH was added to 50 ml of fermentation supernatant in 250 ml glass flask; then the color change of special pH paper indicator on glass flask was monitored by eye to trace the NH_3_ released by NaOH from solution. A spontaneous red-pigmented strain of B3, designated as *Pseudoalteromonas* sp. R3, showed higher LAAO activity, which was confirmed by measuring the concentrations of the produced α-keto acids [11] and H_2_O_2_ (Amplex Red Hydrogen Peroxide/Peroxidase Assay kit, Invitrogen, USA) (data not shown).

Strain R3 or B3 was grown in a marine medium (MM) (0.5% peptone, 0.3% yeast extract and 3% sea salt) for 2 days at 25°C with shaking at 160 rpm. Next, 1 ml of the above culture was added to 250 ml conical flask containing 50 ml MM for fermentation at 28°C with shaking at 160 rpm. After 96 h fermentation, the culture supernatant was harvested with a centrifugation at 8000 rpm for 10 min at 4°C.

### Prussian blue agar assay

Prussian blue agar test was performed according to the report [17] with some modification. Unless otherwise stated, it includes the following steps:(1) dissolve 1.0 g each of FeCl_3_·6H_2_O and potassium hexacyanoferrate (III) in separate 50 ml water to make solutions A and B, respectively; (2) prepare 900 ml MM medium with 2% agar; (3) mix 50 ml solution A with 50 ml solution B to give a 100 ml reddish brown solution C (Prussian brown); (4) pour the 100 ml solution C into 900 ml MM agar medium and adjust pH using NaOH to around 7.5 with resultant formation of a deep green precipitate; (5) autoclave the mixture at 115°C for 30 min and pour into glass Petri dish to make agar plate; (6) make circular wells with diameter 6 mm on plate; (7) for detection, add 50 µl reaction solution to each well and wait for 30 min at room temperature for color change; (8) visualize the Prussian blue forming and measure the blue hole size.

### Stereospecific oxidation of amino acids by LAAO activity

Amino acid was dissolved in 10 ml harvested culture supernatant harboring LAAO activity to have a final concentration of 5 mM. This mixture was incubated for 30 min at 37°C for oxidation reaction. Unless otherwise stated, the mixture pH after oxidation was adjusted to around 7.5. Then, 50 µl of solution was subjected to Prussian blue agar assay. One unit (U) is defined as the amount of enzyme that catalyses the formation of 1 mM H_2_O_2_/h at 37°C.

### α-Keto acid detection

As carbonyl derivatives, a-keto acids was detected by using 2, 4-dinitrophenylhydrazine (DNP) which can react with carbonyl group to generate dinitro-phenylhydrazone with a brown-red color according to the report [11]. Briefly, 500 µl of 5 mM L-amino acid was mixed with 50 µl harvested culture supernatant produced by strain R3 and incubated at 37°C for 60 min. The reaction was terminated by adding 450 µl of 20% trichloroacetic acid and kept at room temperature for 30 min. Next 200 µl of 20 mM DNP was added and the mixture was incubated at room temperature for 15 min. The reaction was terminated after addition of 4 ml of 0.8 M NaOH and further incubation for 15 min at room temperature. Finally, the mixture was centrifuged and the absorbance of the supernatant was measured at 520 nm. The control reaction without L-amino acid was used to subtract the background absorbance.

### Tracking of LAAO activity during fractionation purification

Solid ammonium sulfate was slowly added to 500 ml culture supernatant produced by *Pseudoalteromonas* sp. R3 to give 40% saturation and kept on ice for 5 h. After centrifugation at 10000 rpm for 30 min at 4°C, the supernatant (40% supernatant) and precipitate were collected. The precipitate was dissolved in 10 ml of 0.02 M sodium phosphate buffer (pH 7.4) and dialyzed three times against the same sodium phosphate buffer overnight at 4°C to have a final volume of 20 ml (40% precipitate). The 40% supernatant was further precipitated by slowly adding solid ammonium sulfate to 60% saturation on ice. After incubation on ice for 5 h, the resultant mixture was centrifuged at 10000 rpm for 30 min at 4°C to have the supernatant (60% supernatant) and precipitate. Then, the precipitate was dissolved in 10 ml of 0.02 M sodium phosphate buffer (pH 7.4) and dialyzed three times against the same sodium phosphate buffer overnight to have a final volume of 20 ml (60% precipitate). Finally, 50 µl of each 40% supernatant, 40% precipitate, 60% supernatant and 60% precipitate was used for Prussian blue agar assay to track the LAAO activity.

### SDS-polyacrylamide gel coupled Prussian blue assay

The solutions with LAAO protein were mixed in a 4∶1(v/v) ratio with 4-fold sample loading buffer (1.0 M Tris-HCl, pH 6.8, 10% SDS, 20% ß-mercaptoethanol, 50% glycerol, 1% bromophenol blue). Then, without heating, 20 µl of the resultant mixture was resolved in each well of an SDS-polyacrylamide gel (SDS-PAGE) with a stacking gel of 5% and separation gel of 12%, as described by Laemmli [21]. After electrophoresis and one time wash with water, the resultant gel was directly put on Prussian blue agar for the color development of Prussian blue. Meanwhile, some lanes were released by cutting for Coomassie brilliant blue (CBB) staining according to Kang et al. [22]. After three times wash with distilled water for 5 min, the released CBB stained gel was also directly put together on Prussian blue agar for the color development of Prussian blue. After visualization of Prussian blue, the protein bands near the formed Prussian blue in CBB stained gel were sliced out and directly put together on Prussian blue agar to determine the target LAAO band which caused the formation of Prussian blue. If required, the standard protein ladder and bovine serum albumin (BSA) protein were used for determination of molecular weight of target LAAO.

## Supporting Information

Figure S1 **Measurement of H_2_O_2_ with concentration from 100 µM to 500 µM on Prussian blue agar.** For all detections, 50 µl solutions were added to 6 mm circular well. CK: negative control, without H_2_O_2_.
(TIF)

Figure S2 **Prussian blue agar measurement of H_2_O_2_ produced by** ***Crotalus adamanteus*** **LAAO (caLAAO) activity with L-Leu as substrate (left) and 6.11 mM standard H_2_O_2_ (right).** The diameters of the blue holes were indicated under the holes.
(TIF)

Figure S3 **Tracking of LAAO activity after step-by-step precipitation by solid ammonium sulfate.** The harvested different samples (MM medium, culture supernatant, 40% supernatant, 40% precipitate, 60% supernatant and 60% precipitate) and 5 mM H_2_O_2_ standard solution were separately added to the wells at room temperature for 30 min before photographing. As expected, the MM medium without LAAO enzyme did not give any blue hole, whereas the others all gave the blue holes with different diameters.
(TIF)

Figure S4 **SDS-PAGE coupled in-gel Prussian blue agar assay for determination of LAAO activity.** After electrophoresis, different lanes on SDS-PAGE were sliced out for different treatment and subsequently put together on Prussian blue agar for color development. Lane M: standard protein marker stained with CBB; lane 1: 66 kDa bovine serum albumin (BSA) stained with CBB; Lanes 2 and 3: duplicate 60% precipitate samples from an LAAO producer *Pseudoalteromonas* sp. R3 without CBB staining; lane 4: a replicate of lane-2 and lane-3 with CBB staining; lane 5: the sliced protein bands from a lane-4 replicate as indicated by arrows which are near the formed blue band. The results showed that 60% precipitate sample had only one active protein band which can form Prussian blue band and its molecular weight was around 65 kDa.
(TIF)

Figure S5 **SDS-PAGE coupled in-gel Prussian blue agar assay for detection of caLAAO activity.** After electrophoresis, different lanes on SDS-PAGE were sliced out for different treatment and subsequently put together on Prussian blue agar for color development. Lane 1: R3-LAAO of 60% precipitate without CBB staining; lane 2: caLAAO without CBB staining; lane 3: a lane-2 replicate with CBB staining; lane 4: the sliced protein band from a lane-3 replicate as indicated by arrow. Results showed that caLAAO had only one active protein band and its molecular weight was larger than one of R3-LAAO.
(TIF)

Table S1 **Statistical analysis of dependent variable blue hole diameters from different H_2_O_2_ concentrations by ANOVA.**
(DOC)
