# Supplementary figures and images for: In-Gel Determination of L-Amino Acid Oxidase Activity Based on the Visualization of Prussian Blue-Forming Reaction
Source: PLoS One. 2013 Feb 1;8(2):e55548. doi: 10.1371/journal.pone.0055548 (PMC3562322; doi:10.1371/journal.pone.0055548)

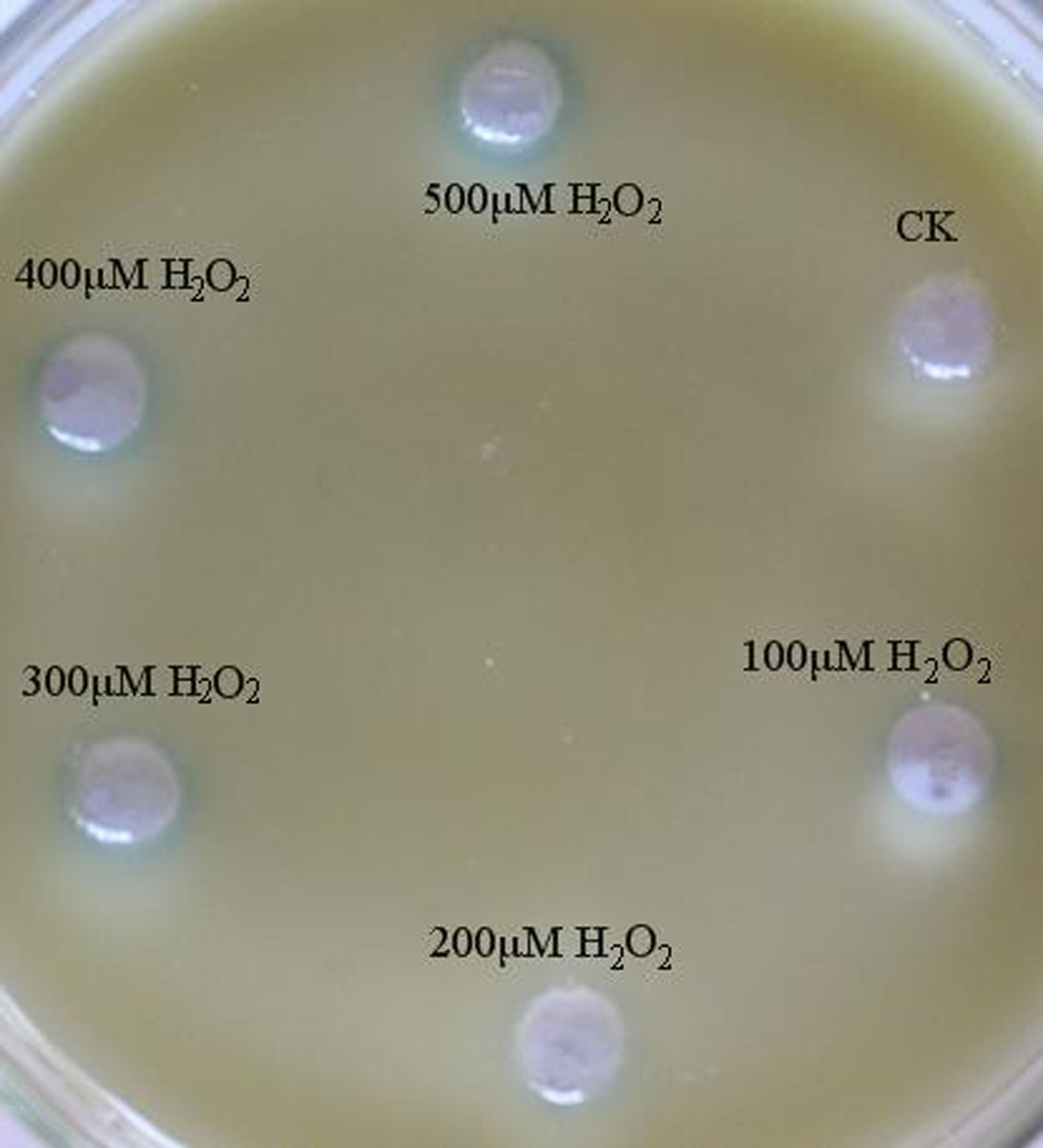

Supplement: Figure S1 — Measurement of H2O2 with concentration from 100 µM to 500 µM on Prussian blue agar. For all detections, 50 µl solutions were added to 6 mm circular well. CK: negative control, without H2O2. (TIF) [file pone.0055548.s001.tif]

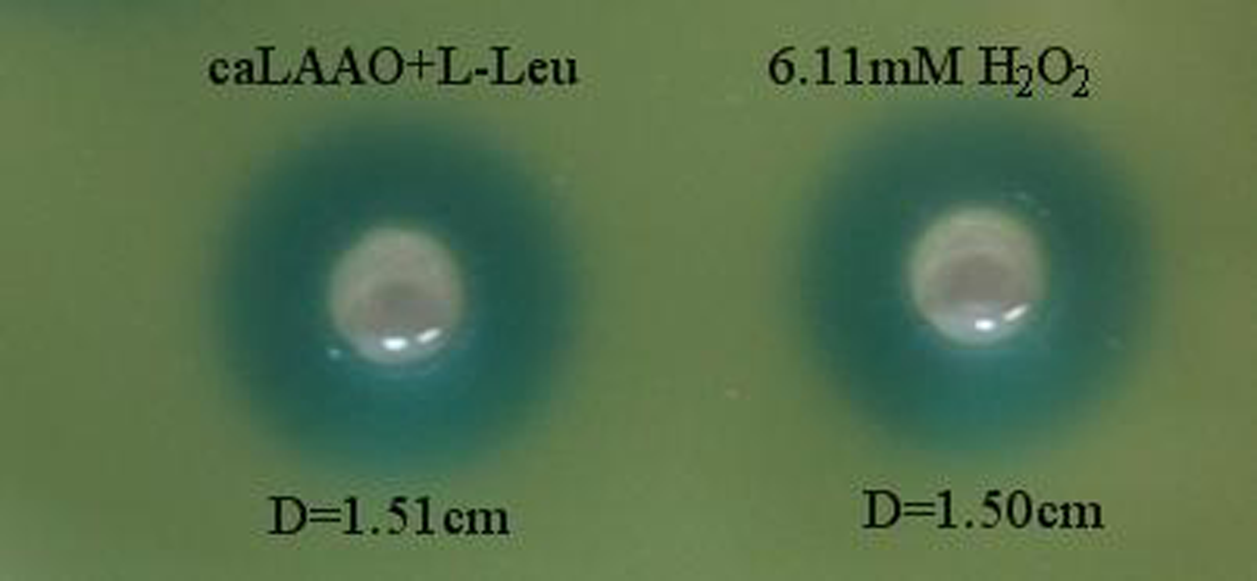

Supplement: Figure S2 — Prussian blue agar measurement of H2O2 produced by Crotalus adamanteus LAAO (caLAAO) activity with L-Leu as substrate (left) and 6.11 mM standard H2O2 (right). The diameters of the blue holes were indicated under the holes. (TIF) [file pone.0055548.s002.tif]

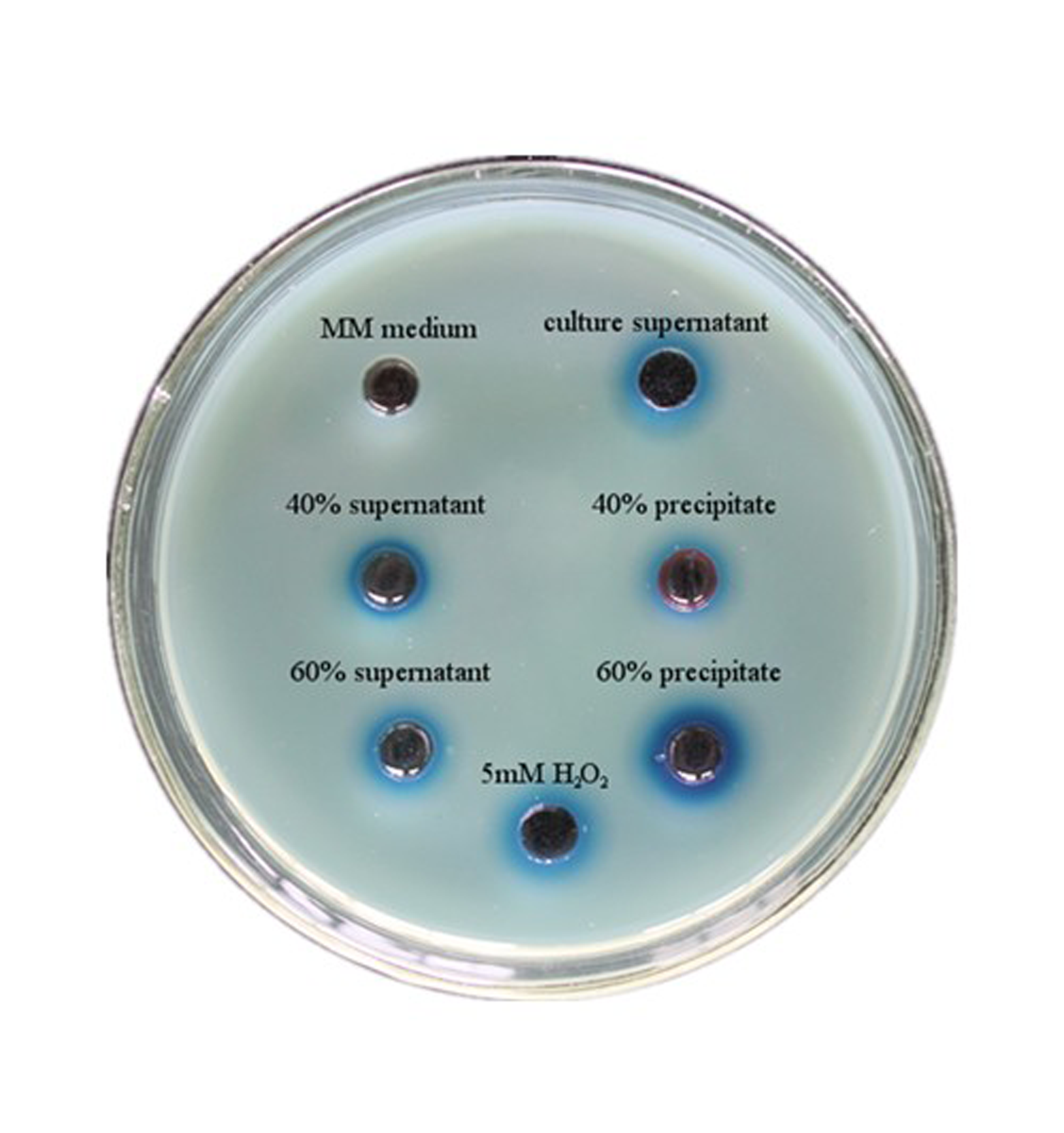

Supplement: Figure S3 — Tracking of LAAO activity after step-by-step precipitation by solid ammonium sulfate. The harvested different samples (MM medium, culture supernatant, 40% supernatant, 40% precipitate, 60% supernatant and 60% precipitate) and 5 mM H2O2 standard solution were separately added to the wells at room temperature for 30 min before photographing. As expected, the MM medium without LAAO enzyme did not give any blue hole, whereas the others all gave the blue holes with different diameters. (TIF) [file pone.0055548.s003.tif]

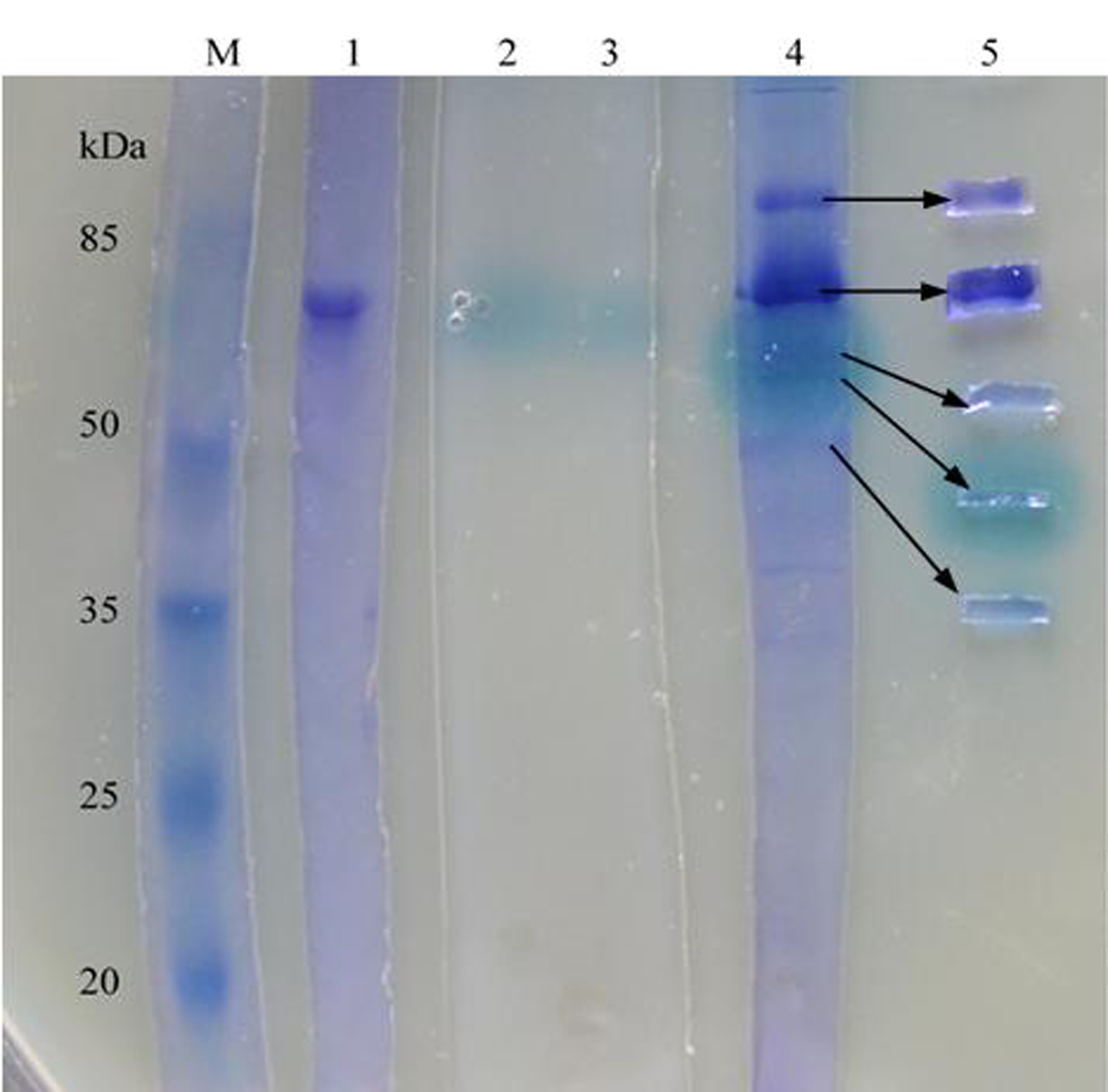

Supplement: Figure S4 — SDS-PAGE coupled in-gel Prussian blue agar assay for determination of LAAO activity. After electrophoresis, different lanes on SDS-PAGE were sliced out for different treatment and subsequently put together on Prussian blue agar for color development. Lane M: standard protein marker stained with CBB; lane 1: 66 kDa bovine serum albumin (BSA) stained with CBB; Lanes 2 and 3: duplicate 60% precipitate samples from an LAAO producer Pseudoalteromonas sp. R3 without CBB staining; lane 4: a replicate of lane-2 and lane-3 with CBB staining; lane 5: the sliced protein bands from a lane-4 replicate as indicated by arrows which are near the formed blue band. The results showed that 60% precipitate sample had only one active protein band which can form Prussian blue band and its molecular weight was around 65 kDa. (TIF) [file pone.0055548.s004.tif]

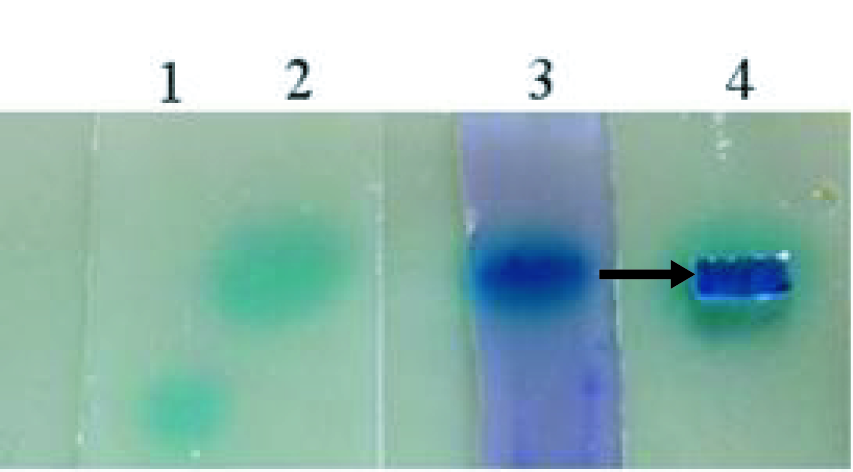

Supplement: Figure S5 — SDS-PAGE coupled in-gel Prussian blue agar assay for detection of caLAAO activity. After electrophoresis, different lanes on SDS-PAGE were sliced out for different treatment and subsequently put together on Prussian blue agar for color development. Lane 1: R3-LAAO of 60% precipitate without CBB staining; lane 2: caLAAO without CBB staining; lane 3: a lane-2 replicate with CBB staining; lane 4: the sliced protein band from a lane-3 replicate as indicated by arrow. Results showed that caLAAO had only one active protein band and its molecular weight was larger than one of R3-LAAO. (TIF) [file pone.0055548.s005.tif]
