# Supplementary material for: In-Gel Determination of L-Amino Acid Oxidase Activity Based on the Visualization of Prussian Blue-Forming Reaction
Source: PLoS One. 2013 Feb 1;8(2):e55548. doi: 10.1371/journal.pone.0055548 (PMC3562322; doi:10.1371/journal.pone.0055548)
Supplement: Table S1 — Statistical analysis of dependent variable blue hole diameters from different H2O2 concentrations by ANOVA. (DOC) [file pone.0055548.s006.doc]

|  | (I)H_2_O_2_ concentration（mM） | (J)H_2_O_2_  concentration（mM） | Mean Difference  (I-J)^a^ | Std. Error | P | 95% Confidence Interval | |
| --- | --- | --- | --- | --- | --- | --- | --- |
|  |  |  |  |  |  | Lower Bound | Upper Bound |
| LSD | 0 | 0.5 | -0.30667*** | 0.01956 | .000 | -0.3478 | -0.2656 |
|  |  | 1 | -0.47667*** | 0.01956 | .000 | -0.5178 | -0.4356 |
|  |  | 2 | -0.63000*** | 0.01956 | .000 | -0.6711 | -0.5889 |
|  |  | 5 | -0.82333*** | 0.01956 | .000 | -0.8644 | -0.7822 |
|  |  | 10 | -1.02000*** | 0.01956 | .000 | -1.0611 | -0.9789 |
|  |  | 20 | -1.23000*** | 0.01956 | .000 | -1.2711 | -1.1889 |
|  |  | 25 | -1.22667*** | 0.01956 | .000 | -1.2678 | -1.1856 |
|  |  | 30 | -1.24333*** | 0.01956 | .000 | -1.2844 | -1.2022 |
|  | 0.5 | 0 | 0.30667*** | 0.01956 | .000 | 0.2656 | 0.3478 |
|  |  | 1 | -0.17000*** | 0.01956 | .000 | -0.2111 | -0.1289 |
|  |  | 2 | -0.32333*** | 0.01956 | .000 | -0.3644 | -0.2822 |
|  |  | 5 | -0.51667*** | 0.01956 | .000 | -0.5578 | -0.4756 |
|  |  | 10 | -0.71333*** | 0.01956 | .000 | -0.7544 | -0.6722 |
|  |  | 20 | -0.92333*** | 0.01956 | .000 | -0.9644 | -0.8822 |
|  |  | 25 | -0.92000*** | 0.01956 | .000 | -0.9611 | -0.8789 |
|  |  | 30 | -0.93667*** | 0.01956 | .000 | -0.9778 | -0.8956 |
|  | 1 | 0 | 0.47667*** | 0.01956 | .000 | 0.4356 | 0.5178 |
|  |  | 0.5 | 0.17000*** | 0.01956 | .000 | 0.1289 | 0.2111 |
|  |  | 2 | -0.15333*** | 0.01956 | .000 | -0.1944 | -0.1122 |
|  |  | 5 | -0.34667*** | 0.01956 | .000 | -0.3878 | -0.3056 |
|  |  | 10 | -0.54333*** | 0.01956 | .000 | -0.5844 | -0.5022 |
|  |  | 20 | -0.75333*** | 0.01956 | .000 | -0.7944 | -0.7122 |
|  |  | 25 | -0.75000*** | 0.01956 | .000 | -0.7911 | -0.7089 |
|  |  | 30 | -0.76667*** | 0.01956 | .000 | -0.8078 | -0.7256 |
|  | 2 | 0 | 0.63000*** | 0.01956 | .000 | 0.5889 | 0.6711 |
|  |  | 0.5 | 0.32333*** | 0.01956 | .000 | 0.2822 | 0.3644 |
|  |  | 1 | 0.15333*** | 0.01956 | .000 | 0.1122 | 0.1944 |
|  |  | 5 | -0.19333*** | 0.01956 | .000 | -0.2344 | -0.1522 |
|  |  | 10 | -0.39000*** | 0.01956 | .000 | -0.4311 | -0.3489 |
|  |  | 20 | -0.60000*** | 0.01956 | .000 | -0.6411 | -0.5589 |
|  |  | 25 | -0.59667*** | 0.01956 | .000 | -0.6378 | -0.5556 |
|  |  | 30 | -0.61333*** | 0.01956 | .000 | -0.6544 | -0.5722 |
|  | 5 | 0 | 0.82333*** | 0.01956 | .000 | 0.7822 | 0.8644 |
|  |  | 0.5 | 0.51667*** | 0.01956 | .000 | 0.4756 | 0.5578 |
|  |  | 1 | 0.34667*** | 0.01956 | .000 | 0.3056 | 0.3878 |
|  |  | 2 | 0.19333*** | 0.01956 | .000 | 0.1522 | 0.2344 |
|  |  | 10 | -0.19667*** | 0.01956 | .000 | -0.2378 | -0.1556 |
|  |  | 20 | -0.40667*** | 0.01956 | .000 | -0.4478 | -0.3656 |
|  |  | 25 | -0.40333*** | 0.01956 | .000 | -0.4444 | -0.3622 |
|  |  | 30 | -0.42000*** | 0.01956 | .000 | -0.4611 | -0.3789 |
|  | 10 | 0 | 1.02000*** | 0.01956 | .000 | 0.9789 | 1.0611 |
|  |  | 0.5 | 0.71333*** | 0.01956 | .000 | 0.6722 | 0.7544 |
|  |  | 1 | 0.54333*** | 0.01956 | .000 | 0.5022 | 0.5844 |
|  |  | 2 | 0.39000*** | 0.01956 | .000 | 0.3489 | 0.4311 |
|  |  | 5 | 0.19667*** | 0.01956 | .000 | 0.1556 | 0.2378 |
|  |  | 20 | -0.21000*** | 0.01956 | .000 | -0.2511 | -0.1689 |
|  |  | 25 | -0.20667*** | 0.01956 | .000 | -0.2478 | -0.1656 |
|  |  | 30 | -0.22333*** | 0.01956 | .000 | -0.2644 | -0.1822 |
|  | 20 | 0 | 1.23000*** | 0.01956 | .000 | 1.1889 | 1.2711 |
|  |  | 0.5 | 0.92333*** | 0.01956 | .000 | 0.8822 | 0.9644 |
|  |  | 1 | 0.75333*** | 0.01956 | .000 | 0.7122 | 0.7944 |
|  |  | 2 | 0.60000*** | 0.01956 | .000 | 0.5589 | 0.6411 |
|  |  | 5 | 0.40667*** | 0.01956 | .000 | 0.3656 | 0.4478 |
|  |  | 10 | 0.21000*** | 0.01956 | .000 | 0.1689 | 0.2511 |
|  |  | 25 | 0.00333 | 0.01956 | .867 | -0.0378 | 0.0444 |
|  |  | 30 | -0.01333 | 0.01956 | .504 | -0.0544 | 0.0278 |
|  | 25 | 0 | 1.22667*** | 0.01956 | .000 | 1.1856 | 1.2678 |
|  |  | 0.5 | 0.92000*** | 0.01956 | .000 | 0.8789 | 0.9611 |
|  |  | 1 | 0.75000*** | 0.01956 | .000 | 0.7089 | 0.7911 |
|  |  | 2 | 0.59667*** | 0.01956 | .000 | 0.5556 | 0.6378 |
|  |  | 5 | 0.40333*** | 0.01956 | .000 | 0.3622 | 0.4444 |
|  |  | 10 | 0.20667*** | 0.01956 | .000 | 0.1656 | 0.2478 |
|  |  | 20 | -0.00333 | 0.01956 | .867 | -0.0444 | 0.0378 |
|  |  | 30 | -0.01667 | 0.01956 | .405 | -0.0578 | 0.0244 |
|  | 30 | 0 | 1.24333*** | 0.01956 | .000 | 1.2022 | 1.2844 |
|  |  | 0.5 | 0.93667*** | 0.01956 | .000 | 0.8956 | 0.9778 |
|  |  | 1 | 0.76667*** | 0.01956 | .000 | 0.7256 | 0.8078 |
|  |  | 2 | 0.61333*** | 0.01956 | .000 | 0.5722 | 0.6544 |
|  |  | 5 | 0.42000*** | 0.01956 | .000 | 0.3789 | 0.4611 |
|  |  | 10 | 0.22333*** | 0.01956 | .000 | 0.1822 | 0.2644 |
|  |  | 20 | 0.01333 | 0.01956 | .504 | -0.0278 | 0.0544 |
|  |  | 25 | 0.01667 | 0.01956 | .405 | -0.0244 | 0.0578 |

^a^The mean difference is not significant at P>0.05 and extremely significant (***) at P<0.001.
